# Supplementary material for: Utility of carotid ultrasound on prediction of 1-year mortality in emergency department patients with neurological deficits: A 10-year population-based cohort study
Source: PLoS One. 2022 Dec 19;17(12):e0277951. doi: 10.1371/journal.pone.0277951 (PMC9762588; doi:10.1371/journal.pone.0277951)

**Supplemental Table 1.** Numbers (%) of different stenosis severity over each segment of carotid artery

**Supplemental Figure 1.** Discrimination of the regression model tested by area under the receiver operating characteristic curve (AUROC) for each outcome (A) 1-year mortality (B) 1-year CV-related mortality (C) 180-day mortality (D) 180-day CV-related mortality (E) 90-day mortality (F) 90-day CV-related mortality

**Supplemental Figure 2.** Using ROC curve and Youden Index (YI) to determine cutoff value of carotid plaque score (CPS) for predicting 1-year mortality. (A) All-cause mortality, best cutoff value: CPS ≥ 6. (B) CV-related mortality, best cutoff value: CPS ≥ 5

| **Supplemental Table 1.** Numbers (%) of different stenosis severity over each segment of carotid artery | | | | | | | |
| --- | --- | --- | --- | --- | --- | --- | --- |
|  | Normal | <30% stenosis | 30-49% stenosis | 50-69% stenosis | 70-99% stenosis | Total occlusion | Previous stenting |
| Right carotid bulb | 1582 (19.9) | 5234 (65.7) | 1099 (13.8) | 41 (0.5) | 5 (0.1) | 0 (0.0) | 16 (0.2) |
| Right CCA | 2721 (34.2) | 4926 (61.9) | 284 (3.6) | 22 (0.3) | 5 (0.1) | 3 (0.0) | 7 (0.1) |
| Right ICA | 4817 (60.5) | 2355 (29.6) | 411 (5.2) | 221 (2.8) | 143 (1.8) | 14 (0.2) | 17 (0.2) |
| Right ECA | 6086 (76.4) | 1495 (18.8) | 326 (4.1) | 52 (0.7)^a^ | | 2 (0.0) | 0 (0.0) |
| Left carotid bulb | 1614 (20.3) | 5285 (66.4) | 1012 (12.7) | 32 (0.4) | 12 (0.2) | 6 (0.1) | 18 (0.2) |
| Left CCA | 2516 (31.6) | 5010 (62.9) | 395 (5.0) | 21 (0.3) | 7 (0.1) | 12 (0.2) | 4 (0.1) |
| Left ICA | 4837 (60.8) | 2347 (29.5) | 395 (5.0) | 199 (2.5) | 132 (1.7) | 51 (0.6) | 16 (0.2) |
| Left ECA | 6453 (81.1) | 1210 (15.2) | 248 (3.1) | 42 (0.6)^a^ | | 8 (0.1) | 0 (0.0) |
| CCA=common carotid artery, ECA=external carotid artery, ICA=internal carotid artery ^a^ 50-69% stenosis and 70-99% stenosis were merged in ECA because high degree stenosis was hard to be distinguished during ECA scanning | | | | | | | |

**Supplemental Figure 1.** Discrimination of the regression model tested by area under the receiver operating characteristic curve (AUROC) for each outcome (A) 1-year mortality (B) 1-year CV-related mortality (C) 180-day mortality (D) 180-day CV-related mortality (E) 90-day mortality (F) 90-day CV-related mortality

CV=cardiovascular


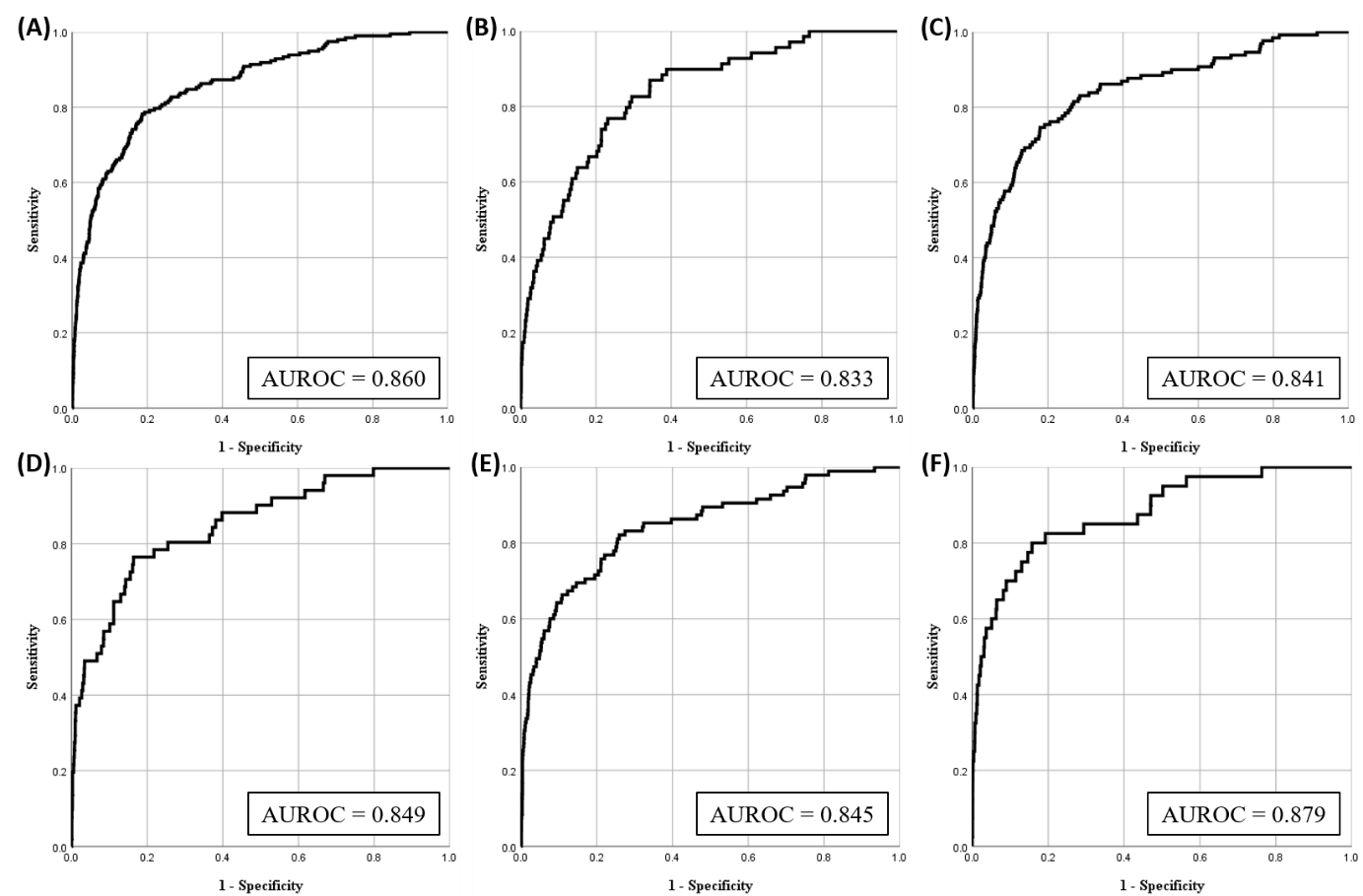


**Supplemental Figure 2.** Using ROC curve and Youden Index (YI) to determine cutoff value of carotid plaque score (CPS) for predicting 1-year mortality. (A) All-cause mortality, best cutoff value: CPS ≥ 6. (B) CV-related mortality, best cutoff value: CPS ≥ 5

AUC=area under curve, CV=cardiovascular, ROC=receiver operating characteristic


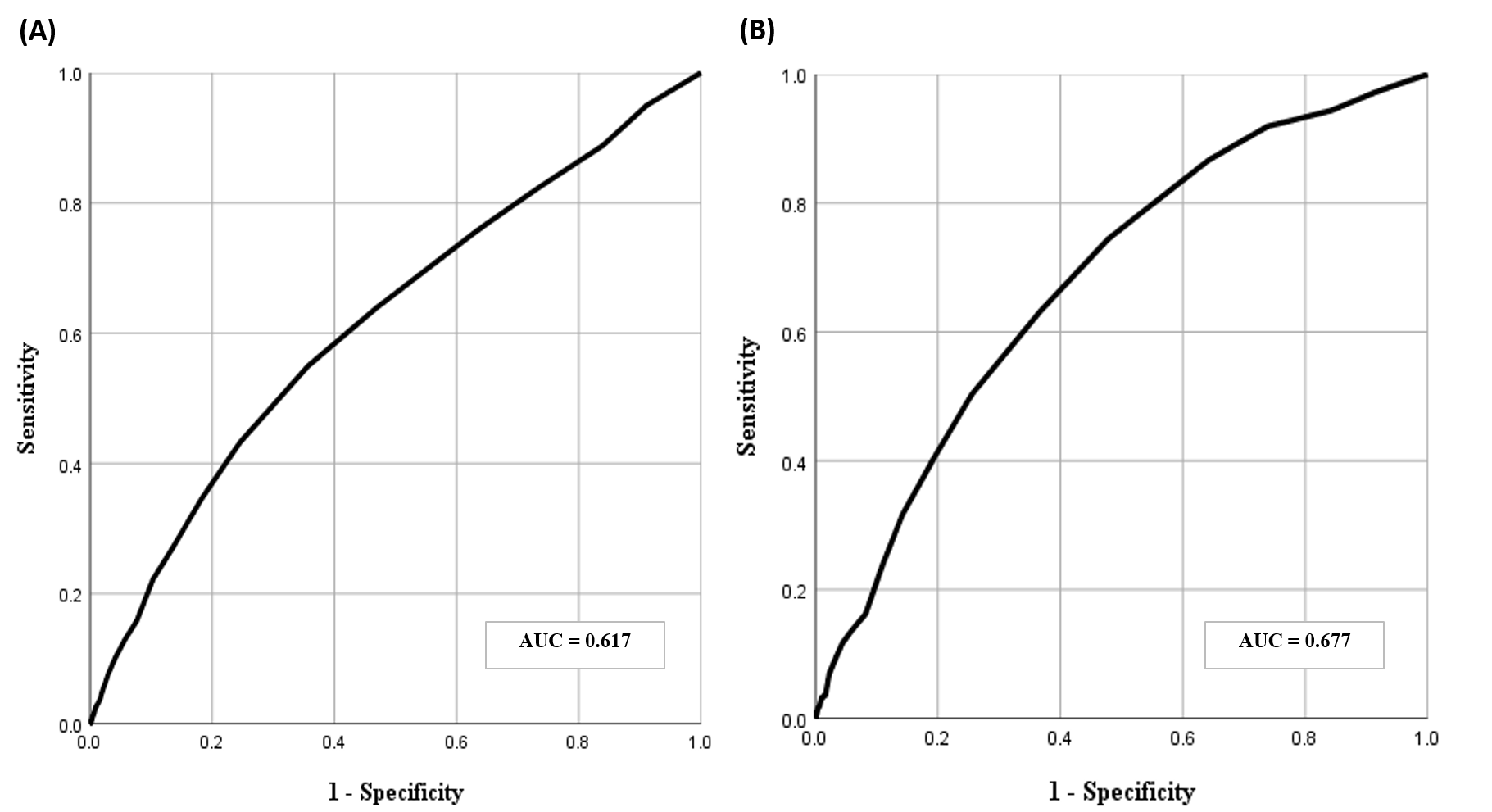

Supplement: S1 File — (DOCX) [file pone.0277951.s002.docx]
